# Supporting Mental Health with Apps: A Systematic Review of Potential and Quality of Implemented Behavior Change Techniques in Mobile Health Applications

**DOI:** 10.3390/ejihpe16010013

**Published:** 2026-01-14

**Authors:** David Leistner, Fabio Richlan

**Affiliations:** 1 Centre for Cognitive Neuroscience, Paris-Lodron-University of Salzburg, 5020 Salzburg, Austria; 2 Department of Psychology, Paris-Lodron-University of Salzburg, 5020 Salzburg, Austria

**Keywords:** applications, apps, behavior change, mental health, mHealth, mobile health, review

## Abstract

The rapid digitalization of healthcare has led to the widespread availability of mobile health (mHealth) applications, including those aimed at mental health and well-being. The present study followed the PRISMA guidelines and systematically reviewed English and/or German mental health apps available in the Google Play Store to evaluate their functional quality and behavior-change potential. It utilized the Mobile App Rating Scale (MARS) to assess app quality, including engagement, functionality, esthetics, and information quality, and the App Behavior Change Scale (ABACUS) to evaluate the potential for behavior change by inclusion of behavior change techniques (BCTs). A total of 77 apps were reviewed, with findings indicating an average functional quality and moderate behavior-change potential, as the reviewed apps only utilized a limited amount of BCTs. Notably, only a small fraction of apps had been evaluated in randomized controlled trials (RCTs). Further analysis showed that MARS and ABACUS scores had limited predictive power regarding app popularity as measured by stars awarded by users and number of user ratings in the Google Play Store. The study highlights the need for more rigorous testing of mHealth apps and suggests that factors beyond those measured by MARS and ABACUS may influence app popularity. In addition to the scientific value, this review provides insights for both users interested in mental health support via apps and developers aiming to enhance the quality and impact of mental health applications.

## 1. Introduction

In a world with ever-increasing digitalization and growing dependence on mobile computing devices such as smartphones, the relevance of these devices for health management becomes increasingly clear. This need has given birth to the rise in the field of mHealth (mobile health) applications, a part of the broader eHealth (electronic health) field (Meier et al., 2013). Such applications aim to help with different aspects of health, such as smoking cessation (or addiction management in general), physical activity, or applications for specific diseases such as diabetes (Bricca et al., 2022; Haskins et al., 2017; Schoeppe et al., 2017). The field of mental health has experienced a surge in applications over the past few years, reflecting its growing prominence (Berrouiguet et al., 2018). With people spending more time on their smartphones, mobile apps can provide an effective tool for delivering health-oriented interventions (Meier et al., 2013).

Smartphone ownership is growing continuously and is estimated to reach 6.2 billion users by 2029 (Global: Number of Smartphone Users 2014–2029|(Statista, n.d.-a)). Younger people, those with higher levels of education and higher incomes are more likely to own a smartphone, according to a report by Pew Research Center (Pew Research Center, 2019). This shows the huge potential for reaching a broad audience at a relatively low cost with targeted interventions (Meier et al., 2013).

The growing number of mental-health-focused apps has yet to receive extended attention from research scientists evaluating the quality of the developed applications. This is specifically important for two groups of people. First, for users, to ensure that the time, effort and even money they spend on these mental health apps brings the intended effects. Second, for the developers of the apps, as they have an interest in designing effective apps that then sell better and can help the users, even leading to certifications for apps, for example, with the GKV Spitzenverband, the nationwide association of health insurance funds in Germany, where apps can be certified to be financed by health insurance providers (GKV Spitzenverband, n.d.).

The present review breaks new ground by assessing the functional quality and behavior change potential of mHealth apps focusing on mental health. As using randomized controlled trials (RCTs) to test the effectiveness of the apps (which would be the gold standard) is impractical for the large number of currently available apps, this review uses a different method, which was proposed by McKay et al. (2019a), by combining the Mobile App Rating Scale (MARS) (S. R. Stoyanov et al., 2015) and the App Behavior Change Scale (ABACUS) (McKay et al., 2019a).

Many mobile applications are short-lived, and search results can change fast. Larsen et al. (2016) showed that on Android, 50% of the search results for the keyword “depression” changed after only 130 days (for “bipolar disorder” this happened after 195 days; for “suicide prevention” even after 115 days). This means that, on average, every 2.9 days one app associated with the keyword “depression” becomes unavailable for download. Due to this fast turnover rate, it is important to assess the quality and potential for behavior change repeatedly. As RCTs, which would be the most effective and reliable form of study, are very time- and effort-consuming, McKay et al. (2019a) argued that reviews using the combination of MARS and ABACUS present a viable option to keep up with the rapid development and at the same time generate a usable and comparable estimate of quality and potential for behavior change (and as such of effectiveness).

### 1.1. Development of the Field of eHealth and mHealth Interventions

Already as early as 2003 there have been calls for standardization and regulation of eHealth interventions by researchers and developers alike. Qualitative interviews conducted with a wide range of different stakeholders—developers, researchers, opinion leaders, project managers, physicians, consumers—showed that there has been a strong wish for consensus and standardization (Ahern et al., 2006). Additionally, one key area was the evaluation methods and challenges that come with it. As mentioned before, RCTs present unique challenges for the eHealth sector. Along these lines the problem of clearly defining and measuring the outcome variables was brought up, as objective markers of behavior change are preferred to subjective assessments by the participants (Ahern et al., 2006).

Studies have compared the effectiveness of mHealth interventions to conventional treatments. For instance, Ben-Zeev et al. (2018) compared a smartphone-delivered intervention called “FOCUS” to a clinic-based group intervention for patients with mental illness. Both programs improved patients’ health significantly, but the mHealth intervention had higher uptake and engagement. This suggests the potential of this specific intervention and mHealth interventions in general. In addition, Rowland et al. (2020) reviewed existing evidence on mHealth. Few apps using behavior change techniques undergo high-quality trials. Apps that digitalize traditional therapy methods, like cognitive behavioral therapy (CBT), show a range of effect sizes, and higher user engagement is associated with improved outcomes. Disease-related education applications/interventions can also yield positive outcomes. However, the evidence is preliminary due to limitations like short time periods, specific populations, and different effect sizes.

Physical movement is an important factor in maintaining mental health. Several studies have shown the link between exercise and mental well-being. This could be observed among children (Biddle et al., 2019; Zhu et al., 2019), as well as adults (Mahindru et al., 2023; Schuch & Vancampfort, 2021). Recent reviews have also shown that digital behavior change interventions have the potential to promote physical activity (Stockwell et al., 2019; Whatnall et al., 2021). To investigate whether this connection is aptly utilized in mental health-oriented apps, we exploratively assessed how many of the observed apps include physical movement into their programs.

### 1.2. Behavior Change and Behavior Change Techniques (BCTs)

Apps that aim to change the behavior of their users profit greatly from including techniques that are already used and evaluated in the literature and practice. A Behavior Change Technique (BCT) is “an observable, replicable, and irreducible component of an intervention designed to alter or redirect causal processes that regulate behavior […]” (Michie et al., 2013).

BCTs include techniques that aim to change the target behavior towards a more desirable one. This can include techniques like goal setting, instructions on how to perform behavior, and feedback on behavior (Michie et al., 2013). As there are a huge number of different techniques that can be used, Michie et al. (2013) created a taxonomy including 93 BCTs grouped into 16 categories that summarize the techniques already used in research and practice. Using such techniques in an app can improve the app’s potential to change the target behavior in the user. This is especially important in apps targeting mental health that have a clear objective and that people use to improve their life.

When working with BCTs it should be kept in mind that different combinations of techniques can work better than others. Dusseldorp et al. (2014) showed that the most effective combinations were the following: *Provide information about behavior–health link* with *Prompt intention formation* (Prompts that help the user to form intentions about future behavior), *Provide information about behavior–health link* with *Provide information on consequences* and *Use of follow-up prompts*. Additionally, some techniques were shown to be ineffective without another technique complementing them; for example, the use of *Provide feedback in performance* without using *Provide instruction* (Dusseldorp et al., 2014).

### 1.3. Existing Research in mHealth

Alslaity et al. (2022) reviewed mHealth apps from the health and wellness categories using the ABACUS and Persuasive System Design (PSD) framework. The PSD model is a framework for analyzing, designing, and evaluating persuasive systems (Oyibo, 2021). Alslaity et al. (2022) developed their own Behavior Change Score (BCS) using items from both ABACUS and PSD. Apps were classified into categories, with journaling apps as the most common, followed by habit-tracking apps and mental health apps. They found that the apps considered used twelve strategies on average. The most-employed strategies were *self-monitoring*, *customization and personalization*, and *reminders*.

McKay et al. (2019b) reviewed apps from five different lifestyle categories (physical activity, healthy eating, smoking and alcohol cessation/reduction, and improved mental well-being) using MARS and ABACUS. The 344 apps received an average score of 2.93 (out of 5) for MARS, and an average score of 7.80 (out of 21) for ABACUS. These values can be interpreted as low-to-moderate functionality (MARS) and a low-to-moderate number of BCTs were included in the apps. The most-often-included BCTs were *practice and rehearsal*, followed by *instructions* and *self-monitoring*. The authors also noted that only one third included the possibility of setting goals. Regarding the low number of BCTs used, the authors point out an opportunity for growth in the apps of the reviewed categories. Additionally, this review served as proof that ABACUS and MARS can successfully be applied for apps of different categories.

Edwards et al. (2016) used a different approach in their review of BCTs in mental health apps. This older review used the BCT Taxonomy v1 by Michie et al. (2013). This taxonomy is a collection and categorization of 93 BCTs collected from the literature. The taxonomy was referenced and included into the creation of the ABACUS (McKay et al., 2019a). Edwards et al. (2016) showed that in their 64 reviewed apps, the BCTs most often used were *self-monitoring* (in 86% of apps), *non-specific reward* (in 82% of apps), and *social support—unspecified* (75% of apps). The median number of BCTs used in the reviewed apps was 14, with a range from 5 to 22 techniques and a negatively skewed distribution. In contrast to other reviews, they also included apps found in the National Health Service (NHS) Health Apps Library, a service which has been closed since December 2021 (NHS England, n.d.), that gave an overview of health apps reviewed and recommended by the NHS.

Antezana et al. (2020) used a similar approach in their review, though they laid their focus on other categories of apps. This review investigated the top-listed health and lifestyle apps for the following categories: physical activity, diet, and sleep. Like Edwards et al. (2016), they used the behavior change techniques taxonomy v1 (Michie et al., 2013) to categorize the BCTs they found in the apps. Their review found that *feedback on outcomes of behavior* was the most common technique (76%), followed by *self-monitoring of outcomes of behavior* (67%), and *social support—unspecified* (60%). Their findings were similar to those of Edwards et al. (2016), suggesting that similar techniques are used over different app categories.

Similar reviews were conducted for apps targeting physical activity. One example is the review by Simões et al. (2018). They used MARS (S. R. Stoyanov et al., 2015) to assess the quality of the apps, and an older variant of the BCTs taxonomy developed by Abraham and Michie (2008). In the 51 reviewed apps, they found 5.5 BCTs on average. This indicates that the apps used few BCTs on average. The most-often-used techniques were *providing feedback on performance* (98%), *self-monitoring of behavior* (98%), and *prompt specific goal setting* (82%). This is again similar to the reviews mentioned before, showing that providing feedback and prompting self-monitoring are popular BCTs for mHealth apps. The total mean MARS score for the reviewed apps was 3.88, indicating that the average quality of the apps was good. This, however, is less surprising considering their sample, as only apps with a user rating of at least four stars (out of five) were included.

This non-exhaustive overview shows that different measures were used to evaluate the BCTs and behavior change potential of mobile apps. One often-used tool is Michie et al. (2013)’s BCTs taxonomy v1, or even an earlier categorization by Abraham and Michie (2008). More recent reviews use ABACUS (McKay et al., 2019a), which aims to make the review process simpler and the results more comparable, as each app then receives one score that indicates the potential for behavior change.

The review of mHealth apps by Bricca et al. (2022) is one example, in which the combination of MARS and ABACUS has been used to assess the quality and potential for behavior change for health applications. The focus of this review was apps targeting specific chronic health conditions such as osteoarthritis, heart conditions, or hypertension. Though the study’s results are not of relevance for the present review due to different scope, it shows the methodological feasibility of using the combination of MARS and ABACUS for reviewing mHealth applications.

### 1.4. The Present Review

The present review investigates apps that market themselves as improving mental wellness and mental health, with a focus on non-clinical applications. This area enjoys growing popularity with users but has received little attention from the research community as of now. Applications in eHealth and mHealth continue to grow, with new applications being continuously developed and published, but seldom evaluated in their effectiveness. Therefore, the present review is aimed at the following questions:

(1) What is the potential for behavior change and the functional quality of mHealth apps aimed at mental well-being and mental health? For evaluation, MARS (S. R. Stoyanov et al., 2015) and ABACUS (McKay et al., 2019a) were used. MARS measures the functional quality of an app concerning engagement, functionality, esthetics, and information. ABACUS assesses the potential for behavior change by measuring the number of BCTs that are used in the app. Based on past research as presented above, we expected that the observed apps would include a small-to-medium number of BCTs (around 10 BCTs on average). The functional quality based on the MARS score was assumed to be average, as a wide range of apps were included.

(2) Can the functional quality and potential for behavior change predict the popularity of the app? It can be assumed that quality and number of BCTs can predict the popularity of the apps. Aspects like esthetics and functionality as measured in the MARS influence the user experience. We expected that a better user experience would result in higher popularity. Higher potential for behavior changes brings higher potential that users reach the intended goal of the app and, therefore, are more satisfied, which, in turn, should result in higher popularity (as measured in stars awarded by users and number of user ratings).

As physical movement is an important part of holistic (mental) health care, we exploratively assess how many of the reviewed apps include physical movement into their content. The combination of MARS and ABACUS has been used in other areas of mHealth applications already, such as physical activity (Schoeppe et al., 2017). This review transfers the methodology of these examples to the topic of mental-health-focused mobile applications.

## 2. Materials and Methods

The present review of mental health applications followed the PRISMA guidelines for systematic reviews (Page et al., 2021b). These guidelines include rules for meta-analysis and systematic (literature) reviews that aim to guarantee a high level of objectivity and reliability. They also aim for maximum transparency, so that the results of the review/analysis can be replicated. The review was not registered.

### 2.1. Inclusion and Exclusion Criteria for Mobile Apps

Inclusion criteria were as follows: applications that aim to improve the user’s mental health and mental well-being. Apps had to be available either in German or English or both. Following the criteria of earlier reviews (Alslaity et al., 2022), only apps with a minimum user rating of three stars, 1000 downloads, and 100 user ratings on the Google Play Store were considered. Although this constraint reduces the variance of the observed apps, the decision was made as potential users will use the ratings as orientation for their choice of app and are unlikely to download apps with a worse rating (Burgers et al., 2016; Oh et al., 2015). Additionally, this procedure reduces the very high number of available applications to a manageable number. These inclusion criteria were set as filters in the Google Play Store search, and therefore it is impossible to state the exact number of apps that did not meet these criteria. Estimates on the total number of mental health apps globally available in the Google Play Store range from 10,000 to 20,000 (Carrouel et al., 2022; Mehrotra et al., 2025).

Exclusion criteria were as follows: apps for clinical applications; apps for a specific diagnosis; apps for specific areas of health such as smoking cessation, alcohol consumption reduction, diet, meditation, and nutrition. All these areas have been excluded since, firstly, some have been the subject of prior study (Colbert et al., 2020; Coughlin et al., 2015; Haskins et al., 2017; Roquet & Sas, 2018), and, secondly, they deserve a separate look due to the highly specialized field of application. This review focused on apps that aim to improve mental health in general.

The search for apps was conducted in the Google Play Store from 1st of March to 12th of March 2024. Apps were searched using the Play Store Web Page (https://play.google.com/store/apps) (accessed on 12 March 2024) and then cross-referenced with the search results on the Google Play Store App on an Android Device (Huawei P20 Pro, Huawei Technologies Co., Ltd., Shenzhen, China). The review only includes the user ratings made for the category phone, and omitted those for tablet, car, and smartwatch, as the phone is the primary device on which people will use the apps. Also, this article refers to the mean of the user ratings found on the webpage and in the app. This is because the Google Play Store only displays user ratings by people with a similar phone and in a similar region as the user (in our case, we used the German Play Store), while on the webpage (Google LLC, n.d.) the user ratings from the United States of America were displayed. By averaging the user ratings from Germany and the US, a mean user rating was achieved that approximates the popularity of the app for the western developed countries. There is an option to choose the European Union as localization on the Google Play Store; however, the search function on that version does not work properly so we decided to use the US Version. Global user ratings were not available to the authors, even after a request to Google.

This review primarily focuses on freely accessible content, since users are most likely to pick free apps over paid versions (Dogruel et al., 2015). Apps were extracted with a keyword search, using the following keywords: mental health, mental well-being, CBT, social connection, anxiety, well-being, relaxation, mindfulness, stress, mood, emotional intelligence, empathy, loneliness, wellness, resilience, and resilient. These keywords were adopted from reviews by McKay et al. (2019b), S. R. Stoyanov et al. (2015), and Alslaity et al. (2022) and complemented with new keywords. All keywords were searched in English and in German. The complete list is available in the Supplementary Material SI.

### 2.2. App Identification and Selection Process

**Figure 1:**
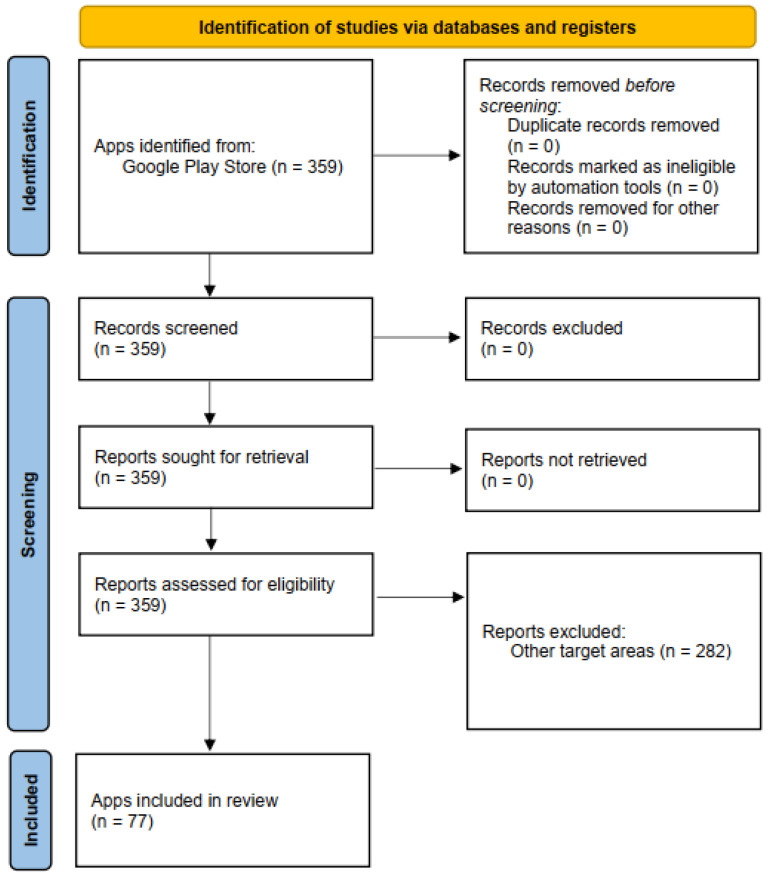
PRISMA 2020 flow diagram.

In a first step, the Google Play Store was searched for the previously defined keywords. Apps that by name and description fit the inclusion criteria were included. As mentioned before, the search was conducted using the web page of the Google Play store, as well as the Google Play Store App on an Android device. In this first round, 359 apps were identified using a rough screening of name and description. In a second round, the identified apps were checked against all inclusion and exclusion criteria, resulting in a total of 77 apps that were included in the review (see Figure 1). These apps were consequently downloaded on a mobile phone and tested before rating. If needed, accounts were created using a dummy e-mail address. The apps were tested over 6 weeks in June and July 2024. On average, each app was tested for half an hour to one hour on one specific day, then rated on MARS and ABACUS by the first author.

### 2.3. Analysis

The apps were rated using MARS (S. R. Stoyanov et al., 2015) to review the overall quality of the app, as well as ABACUS (McKay et al., 2019a) to review the potential for behavior change. Additionally, descriptive statistics were computed to describe the sample.

MARS (S. R. Stoyanov et al., 2015) contains four sections describing different aspects of an application. Section A evaluates the engagement the app offers, section B the functionality, section C the esthetics, and section D the information contained in the app. Mean scores (and standard deviations) for each category as well as the total mean score were computed and compared for each app by the first author. Both authors have several years of professional experience working with mHealth apps at research institutions. In addition, the first author—who evaluated the apps—has been trained for the use of MARS with the training video provided by the authors of the scale, retrieved from YouTube (S. Stoyanov, 2016). In previous studies, MARS displayed a high level of internal consistency (*Cronbach’s alpha* = 0.78) and fair interrater reliability (*two-way mixed ICC* = 0.57, 95% CI = 0.41–0.69) (S. R. Stoyanov et al., 2015).

For ABACUS (McKay et al., 2019a), the apps were rated with a score of 1 (feature exists) or 0 (feature does not exist) for each item, totaling in a possible total score of 21. Mean scores for the four categories (*knowledge and information*, *goals and planning*, *feedback and monitoring*, *actions*), as well as a total mean score were computed and compared between the apps. In earlier studies, the scale showed high interrater reliability (*two-way mixed ICC* = 0.91, 95% CI = 0.81–0.97) and high internal consistency (*Cronbach’s alpha* = 0.93).

In the next step, a regression model was computed to assess whether the MARS (functionality) and ABACUS (behavior change potential) scores could predict the user ratings of the apps (popularity). A second regression model includes the sub/scale scores instead of the total scores of both scales to gain insight into which aspects are more relevant for predicting the popularity of an application. Exploratively, we assessed how many apps included movement into their content, as an indication of how many apps followed a more holistic approach to mental health.

## 3. Results

### 3.1. Overview of the Apps

In total, 77 apps were included in the review. The majority (85.7%) of these apps were from the category *Health and Fitness* in the Google Play Store (*n* = 66). The other apps were from the categories *Lifestyle* (6.5%, *n* = 5), *Medical* (5.2%, *n* = 4), and one each in the categories *Productivity* and *Simulation*.

**Figure 2:**
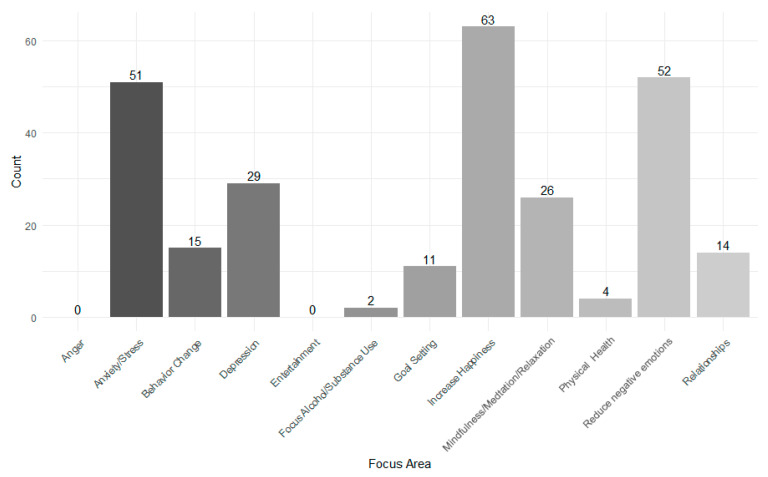
Focus areas of the apps.

The apps had different focus areas within mental health. The most common focus was to *Increase Happiness* (81.8%), followed by *Reduce Negative Emotions* (67.5%), and *Anxiety/Stress* (66.2%). None of the reviewed apps focused on the categories *Anger* or *Entertainment* (see Figure 2). Additional focus areas from the category *Other* (not shown in Figure 2) included: *Sleep, Psychosis, and Compulsions* (one app) and *Sexuality* (one app). Only 13.0% of the apps included physical exercise in any form (*n* = 10), at the minimum prompts to exercise, up to concrete exercise instructions.

**Figure 3:**
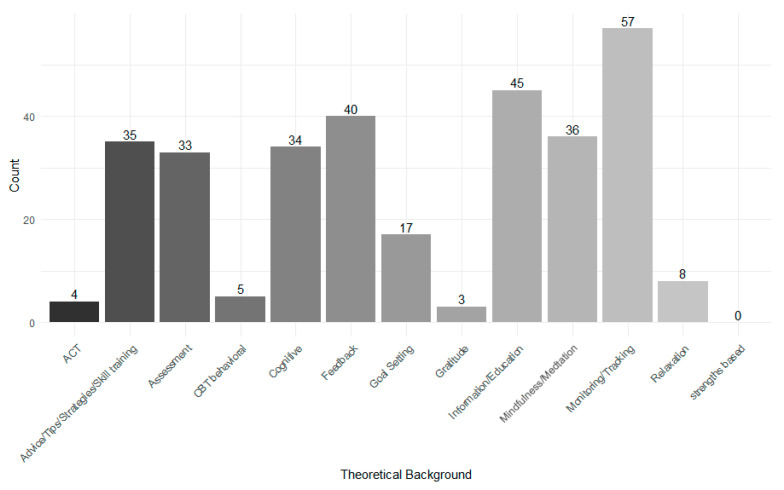
Theoretical background used in the apps.

Regarding their theoretical background, the apps showed a variety of theories used. The most common was *Monitoring/Tracking* (74.0%), followed by *Information/Education* (58.4%), and *Feedback* (52.0%). No app was developed on a *Strengths-Based* background. More details can be found in Figure 3. Additionally, seven apps were categorized in *Other* (not shown in Figure 3) and used *Dialectic Behavior Therapy* (DBT) as theoretical background. Two apps were based on *Positive Psychology*, and one each on *Hypnotherapy*, *Metacognitive Training*, *DARE Training for Anxiety based on MBSC, MBSR and ACT*, *Depth Psychology*, *Eye Movement Interventions*, and *Jungian Psychology*.

Regarding language, only three apps (3.9%) were available in German only. The majority (*n* = 54) were only available in English (70.1%), while 20 apps offered both languages (26.0%).

The mean user rating in the Google Play Store of the selected apps was *M* = 4.33 (*SD* = 0.32, range: 3.30–4.85). The user ratings in the German Google Play Store and the US Google Play Store were averaged to gain an estimation of the average user rating on the western parts of the world, as the Google Play Store does not provide an option to view the global or even European average user ratings of the apps. A total of 21 (27.3%) apps did not have a German user rating available, while only 3 (3.9%) did not have a US user rating. This study included a wide range of apps, with the smallest having only 114 user ratings, whereas the largest came to 547,444 user ratings.

It was found that 63 apps (81.8%) were aimed at a general audience, while only 14 (18.2%) were specifically aimed at adults. An overwhelming 70 out of 77 (90.9%) of apps were supplied by a commercial provider, while 6 (7.8%) were offered by NGOs, and only one (1.3%) was offered by a government. A complete list of the evaluated apps including their MARS and ABACUS scores can be found in Supplementary Material SIV.

**Figure 4:**
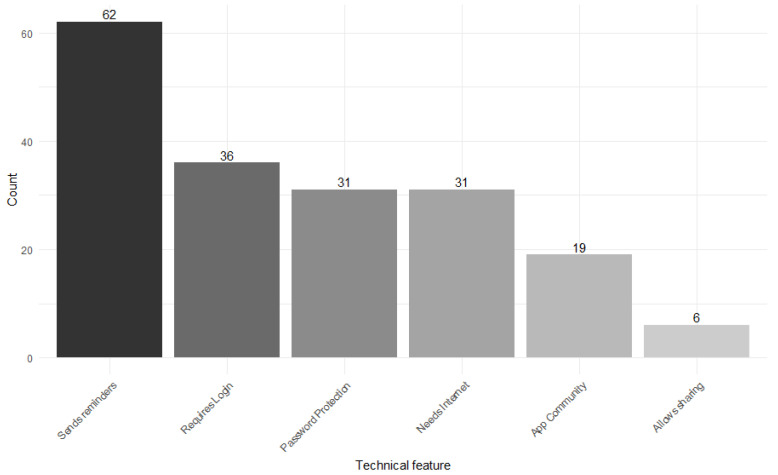
Technical features used in the apps as assessed by MARS.

MARS allows us to capture important technical features of the tested app that are assumed to be important for quality assessment. Regarding these technical features, the most common was *Sending Reminders* (80.5%, *n* = 62) while only six apps offered the possibility to directly *Share* the behavior prompted by the app on social media (7.8%). Thirty-six of the apps required a *Login* to use the app (46.8%), which can add another barrier for potential users. For more details see Figure 4.

### 3.2. MARS—Mobile App Rating Scale

The apps considered in this review were of average functional quality with a mean total score of *M* = 3.69 (*SD* = 0.373, range: 2.79–4.50) on the MARS. The mean score for Section A (*Engagement*) is similar with *M* = 3.67 (*SD* = 0.545, range: 2.40–4.60). This section describes how fun, interesting, customizable, interactive, and well-targeted to an audience the app is. The mean score for section B (*Functionality*) was slightly higher, *M* = 3.81 (*SD* = 0.379, range: 2.75–4.50). This section describes how well an app functions, how easy it is to learn and navigate, as well as the flow logic and gestural design of the app. The mean score for section C (*Esthetics*) was similar with *M* = 3.86 (*SD* = 0.534, range: 2.67–5.00). Section C is concerned with graphical design, overall visual appeal, color schemes and stylistic consistency. The mean score for section D (*Information*) is the lowest out of all sections with *M* = 3.42 (*SD* = 0.515, range: 1.50–4.33). This section evaluates whether the app contains high-quality information. Section E contains questions about *Subjective Quality*. This section was even lower than section D with *M* = 2.99 (*SD* = 0.846, range. 1.25–4.75) but is not included in the total mean MARS score. It represents a subjective rating of the reviewer in terms of whether the app is worth recommending, stimulates repeat use, and provides overall satisfaction. See the detailed scores in Table 1 below.

Only 8 of 77 apps (10.4%) have been tested with an evaluation study of any kind. Four apps (i.e., Betwixt—The Mental Health Game, COGITO Neustart, MKT, Intellect: Create A Better You, and MindDoc: Mental Health Support) have been trialed in RCTs showing positive results (Bruhns et al., 2021, 2023; Kerber et al., 2023; Lüdtke et al., 2018; Moritz et al., 2024; Ong & Sündermann, 2022; Toh et al., 2022; Yokomitsu et al., 2025), and another two apps (i.e., The Self Compassion App, Youper—CBT Chatbot) have been trialed in studies that are not RCTs but with positive results (Beaumont et al., 2025; Mehta et al., 2021). The remaining two fall under the category “App has been trialed (e.g., acceptability, usability, satisfaction ratings) and has partially positive outcomes in studies that are not RCTs, or there is little or no contradictory evidence” (VOS Mental health, Mind reset—Just 2 min a day). Note that many apps displayed “X number of downloads” or “X number of satisfied users” without quoting a source. These apps were categorized as having not been tested, as it was unclear how these numbers were found. For more detailed insights into the results of the MARS subscales, see the Supplementary Material SII. The best 15 apps according to their MARS score can be seen in Table 2.

### 3.3. ABACUS—App Behavior Change Scale

On average, the apps included around nine BCTs (*M* = 9.31, *SD* = 2.86, range: 2–16). The app with the lowest number (Anxiety Relief Apps and Hypnosis) had two techniques, while the highest number were 16 techniques in one app (VOS Mental health, Fabulous Daily Routine Planner). The techniques from area 2—*Goals and Planning*—were used the fewest (see Table 3).

The most-often-used technique for behavior changes in the apps, according to the results of the ABACUS, was *allows self-monitoring of behavior* (*n* = 74), followed by *encouraging positive habit formation* (*n* = 73) and *instruction on how to perform behavior* (*n* = 71). The least-used techniques were all from part *4—Actions*. *Assistance with distraction or avoidance* (*n* = 2) and *provide opportunities to plan for barriers* (*n* = 2) were least used, followed by *assist with or suggest restructuring of physical environment* (*n* = 4). The possibility to *set goals* is offered in only 31.2% (*n* = 24) of the apps. A detailed overview of how many apps used which technique is provided in Supplementary Material SIII.

The 15 apps with the highest ABACUS score can be seen in Table 4. A complete list of the evaluated apps with the respective MARS and ABACUS scores can be found in Supplementary Material SIV.

### 3.4. Predicting App Popularity (Stars Awarded by Users) with the MARS and ABACUS Scores

To answer the question whether the functional quality (MARS) and the potential for behavior change (ABACUS) could predict the popularity of the apps (measured in stars awarded by the users in the Google Play Store), a multiple regression model was used.

**Figure 5:**
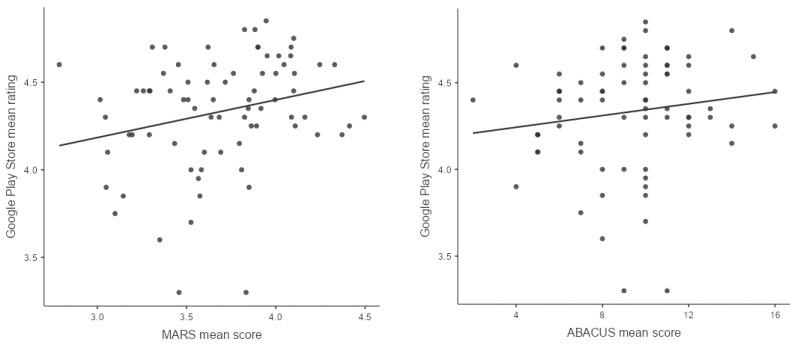
Residual plots for stars awarded by users in the Google Play Store and MARS and ABACUS mean scores, respectively.

Data followed a linear trend, though not strong (see Figure 5). There seemed to be a few outliers, which are identified in the next step.

Using Cook’s distance, observation numbers 6, 12 and 38 could be identified as outliers. Case 6 (Amobear: Mood Tracker, Journal) came to our attention as, despite a low-to-moderate MARS score (2.78), the app received a high mean rating by the users (4.60). This might reflect individual taste and may be an artifact of the limitation that only one researcher reviewed the apps. Case 12 (Awarefy—CBT & AI Therapy) received a moderate-to-high score in the MARS (3.83) and included a fair amount of BCTs, as reflected in the ABACUS score of 11. The mean user rating this app received by the public (3.30), however, was on the lower end of the spectrum we reviewed in this study. A similar pattern held true for case 38 (I’m Fine: Mental Health Guide), which received a low user rating of 3.30 despite an average ABACUS score of 9. After a thorough review to ensure that no mistake had been made in the ratings of the apps, it was decided that those cases should be included despite being outliers, as they possibly delivered valuable information about the relationship between mean user ratings and the more objective MARS and ABACUS scores. The Durbin–Watson Test for autocorrelation showed no significant result (*autocorrelation* = 0.07, *DW* = 1.83, *p* = 0.41), indicating that independent errors could be assumed.

Even though the mean scores of MARS and ABACUS showed a statistically significant correlation (*r* = 0.719, *p* < 0.001), the Variance Inflation Factor (*VIF* = 2.07) and Tolerance Check (*TO* = 0.48) showed no problematic collinearity between the two predictors. The Shapiro–Wilk test for normality was significant (*W* = 0.939, *p* = 0.001), indicating that the data were not normally distributed.

In total, the model with the two variables MARS and ABACUS could only account for 6.35% of variance (*R*^2^ = 0.0635). As seen in Table 5, the overall model test showed that our model could not significantly predict the mean user ratings of the apps using MARS and ABACUS scores.

A detailed look into the model coefficients showed that only the mean score of the MARS exhibited a tendency to predict the mean user rating of an app, *b* = 0.25, *t*(74) = 1.80, *p* = 0.076 (Table 6).

The low amount of variance explained by the MARS and ABACUS scores indicates that popularity as measured via the app user ratings is influenced by other factors that are not included in the two scales. The MARS and ABACUS scores could not sufficiently predict the user ratings of the apps on the Google Play Store. This means that neither the functional quality (as measured with the MARS) nor the number of BCTs (ABACUS score) could predict the popularity of an app (measured in stars awarded by the users in the Google Play Store).

### 3.5. Predicting App Popularity (Number of User Ratings) with MARS and ABACUS Scores

**Figure 6:**
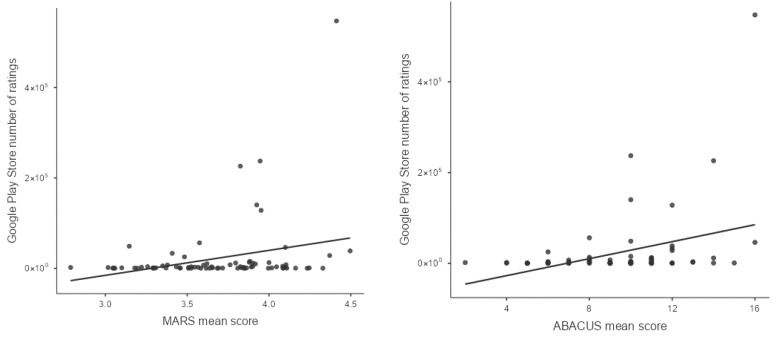
Residual plots for number of Google Play Store user ratings and MARS and ABACUS mean scores, respectively.

As the Google Play Store gives no option to view the exact number of downloads (only a very rough rounded estimate) the number of user ratings was used as an estimate of the number of downloads. The following are the results of the multiple regression model used to predict the popularity of an app (as measured in number of user ratings, as a rough estimate for how often the app was downloaded) using again the MARS and ABACUS scores. The data followed a linear trend, as seen in the following residual plots (Figure 6).

Using cook’s distance, the visible outlier could be identified as case number 32 “Fabulous Daily Routine Planner”. This app springs to attention as it had by far the highest number of user ratings (547,444 ratings) compared to the apps with the second-to-most user ratings, “Cingulo—Mental Wellness” with 237,279 ratings. Additionally, the app had the second-highest MARS total score (4.41) and the highest ABACUS score (16). As no measurement error could be assumed, however, it would be wrong to exclude this case from the analysis. The Durbin–Watson test for autocorrelation showed no significant result (*autocorrelation* < −0.01, *DW* = 2.01, *p* = 0.87), indicating that independent errors could be assumed.

Similarly to the first regression analysis, the Variance Inflation Factor (*VIF* = 2.07) and Tolerance Check (*TO* = 0.48) showed no problematic collinearity between the two predictors. The Shapiro–Wilk test for normality was significant (*W* = 0.569, *p* < 0.001), indicating that the data were not normally distributed. The distribution stemmed from the rapid increase in the number of user ratings in the five most popular apps, where the number of ratings jumped from 56,364 (BetterMe: Mental Health) to 547,444 (Fabulous Daily Routine Planner).

In total, the model could explain 13.2% of the variance (*R*^2^ = 0.132). As seen in Table 7, the overall model test showed that our model could significantly predict the number of user ratings of the apps using MARS and ABACUS scores.

A detailed look into the model coefficients showed that only the mean score of the ABACUS could significantly predict the number of user ratings of an app, *b* = 8669, *t*(74) = 2.14, *p* = 0.035 (Table 8).

This result expands the results of the first regression analysis. While the ABACUS score could significantly predict the number of user ratings, the overall model fit is limited. That is, both scales (MARS and ABACUS) continue to explain little of the variance in the number of user ratings on Google Play Store. This only strengthens the assumption that other factors besides the functional quality (measured with the MARS) and the potential for behavior change (i.e., number of BCTs as measured with the ABACUS) influence the popularity of an app.

## 4. Discussion

The goal of this review was to assess the state of mental-health-focused apps in the Google Play Store and to evaluate their functional quality and potential for behavior change using the Mobile App Rating Scale (MARS) (S. R. Stoyanov et al., 2015) and the App Behavior Change Scale (ABACUS) (McKay et al., 2019a). In total, 77 apps have been reviewed. The observed apps showed an average mean MARS score and moderate ABACUS score.

A surprisingly low number of apps have been subject to RCT for effectiveness (11.7%). Though many more apps claim to be evidence-based or at least created with expertise (75.3%), few go through the extra effort to put this claimed effectiveness to the test. Evidence-based effectiveness should be of higher priority as mHealth services become more prevalent and a viable option for clients to improve their mental health. Especially if such services should be covered by health insurance, they must be thoroughly evaluated, as, for example, in Germany (GKV Spitzenverband, n.d.).

The apps observed used a wide range of BCTs. Including a higher number and a wider variety of said BCTs helps improve the chances of interventions in the app showing the intended effects. On average, the apps used more BCTs than in previous reviews such as by Alslaity et al. (2022) or Simões et al. (2018). Specifically, Alslaity et al. (2022) investigated general health and wellness apps and found that many apps had multiple implementations of different BCTs. The most employed strategies were *self-monitoring*, *customize and personalize*, and *reminders*. In addition, there was a positive correlation between app popularity and behavior change scores. In contrast, Simões et al. (2018)—focusing on physical activity apps—reported an average number of 5.5 BCTs per app. The most frequently used BCTs were *provide feedback on performance* and *prompt self-monitoring of behavior*.

These differences could be an artifact of the sample, but hint towards a positive trend, as including more techniques furthers the chances of effectively changing behavior. In the apps included in the present review, BCTs from the group *Goals and Planning* were the most often used. Among the individual techniques, *self-monitoring behavior*, *encouraging positive habit formation*, and *instruction on how to perform behavior* were most often used.

The least-used techniques were *assistance with distraction or avoidance*, *providing opportunities to plan for barriers*, and *assisting with or suggesting restructuring of physical environment*. Apart from the most-often-used techniques, many techniques were only used by around 20 apps. This shows that there is considerable untapped potential left for the developers to improve their apps by including a wider array of BCTs.

Only around one third of the apps allowed the users to *set goals* themselves. This technique should be used more extensively, as it is easy to implement and has been shown to have a positive effect on behavior change, especially if the goal is difficult, set publicly, and is a group goal (Epton et al., 2017). In addition, goal setting is one of the most frequently used and effective interventions in the sports context (Williamson et al., 2022). In contrast, almost every app allowed the users to *self-monitor their behavior*. This often took the form of a diary or logbook entry (or similar).

The results of the regression model indicate that the variables measured in MARS and ABACUS could not predict the popularity of the app (as measured with the stars awarded by users in the Google Play Store). This result was extended by a second regression analysis, where the number of user ratings was used as a proxy for the number of downloads (as the number of downloads cannot be seen in Google Play Store). The MARS and ABACUS scores could explain more variance in the number of user ratings but only the ABACUS mean score was a significant predictor. Thus, the potential for behavior change seems to have a larger impact than the functional quality of an app on number of user ratings. Taken together, the results indicate that there are other factors impacting whether users like/rate an app or not.

Research has shown that variables like the amount of money spent on marketing (Oh et al., 2015) and the amount of code in an app (which equals amount of content) do have a strong influence on the popularity of an app. Additionally, it is reasonable to assume that app popularity is also influenced by long-term effectiveness, which could not be measured with the methods used in this review. Additionally, it must be kept in mind that the stars awarded by users and number of user ratings are only estimates of the popularity of an app. Applications often prompt users to rate the app after a relatively short time of use, which might not be enough for the users to observe positive effects on their mental health. Furthermore, it does not reflect how long the users continue to use the app, and whether the app achieved its goal after continued use.

Science-based development of especially (mental-) health-focused applications will become increasingly important as health insurance companies start to acknowledge apps as a viable means to deliver health interventions. In Germany, applications can already be certified, and then be administered as interventions by medical doctors, while the health insurance pays for the pro version of the app. The apps, however, need to go through a rigorous testing phase, proving their effectiveness with at least one RCT (GKV Spitzenverband, n.d.).

### 4.1. Limitations

The MARS and ABACUS ratings were conducted by a single researcher (i.e., the first author), carrying the risk of bias. Both scales, however, showed fair interrater reliability in previous studies (McKay et al., 2019a; S. R. Stoyanov et al., 2015), and, therefore, it is reasonable to assume that they are reliable even when carried out by a single person. The first author—who evaluated the apps—has been trained for the use of the MARS with the training video provided by the authors of the scale. In addition, both authors have several years of professional experience working with mHealth apps at research institutions. Nevertheless, future studies might consider including several raters to reliably assess the ratings.

Another limitation stems from the nature of the user ratings in the Google Play Store. As only personalized user ratings are displayed (concerning location and type of device), the selection process can become a hurdle. This was in part mitigated by searching the German as well as the American Play Store, and averaging the user ratings, but would yield different results depending on the localization of the researcher. Using a dedicated market research tool could solve this issue, as those have access to more detailed statistics on the apps (including actual usage). In sum, it is important to remember that the app selection and the popularity of the apps might differ from country to country and any results should only be generalized very cautiously.

The decision to use only Android apps was made for the following reasons: firstly, Android as an operating system holds a market share of 70% worldwide, compared to 30% for Apple iOS (Mobile OS Market Share Worldwide 2009–2023|(Statista, n.d.-b)). Secondly, the Apple App Store gives developers the opportunity to reset user ratings for each new version of the app. This leads to apps having significantly fewer user ratings (as low as 0 ratings for apps that have several thousand for their Android version). This in combination with the smaller user group of Apple devices makes it nearly impossible to create a usable cut-off value to decide which apps to include into the review. Thirdly, and probably for the aforementioned reasons, a similar review by Alslaity et al. (2022) showed that of their 70 included apps, not one was Apple iOS-exclusive. The present review used a similar cut-off value of three stars (out of five) user ratings, following Alsaitys et al.’s approach. For these reasons, this review is focused only on apps available in the Google Play Store, as the work by Alslaity et al. (2022) showed that there is no additional information gained by including the Apple App Store.

Furthermore, this review focused solely on freely accessible content, meaning that some apps may score better once the paid content is unlocked. The present approach seemed feasible, as research has shown that users tend to prefer free apps (Dogruel et al., 2015). Future studies could include the paid features in their analysis, as many of the reviewed apps had a substantial number of their features behind a paywall.

In addition, the cut-off value of 3.00 for the user ratings in the Google Play Store artificially reduced the variance of included apps and may have distorted the results of the regression analysis. If all apps had been included in the review, perhaps the MARS and ABACUS scores could have predicted the user ratings of the apps more consistently. While being a reasonable decision considering economic and practical reasons (i.e., users are unlikely to prefer low-rated apps over high-rated apps), future research could aim to include the complete range of apps to limit bias.

Finally, each app was only tested for a limited amount of time. To obtain a deeper insight into exact effectiveness, longitudinal studies with a limited number of apps should also be considered. As McKay et al. (2019a) rightly argued, the combination of MARS and ABACUS can deliver a reasonable estimate of the quality of apps, which is important in the fast-changing world of mobile applications. To obtain a detailed account of the effectiveness, however, RCTs are needed that due to the high cost can only investigate a few apps at a time.

### 4.2. Recommendations for Users

Choosing the right mental health app largely depends on the momentary need of the user. Many of the reviewed apps are specialized in some ways. Some are geared mainly towards emotion and habit tracking, some towards journaling, while some focus more on meditation or psychoeducation.

Regarding the targeted mental health issue there are also differences. Some focus more on anxiety and stress, some on depression, some on other habits and behavior change. A few of the apps follow a more holistic approach in target issues and methods to be better able to adapt to the needs of the user.

Only very few of the reviewed apps (13%) include physical exercises in their repertoire. Note, however, that apps specialized in yoga and meditation, without explicit focus on mental health, were excluded from this review. These apps would eventually have a positive impact on the user’s mental health but do not explicitly claim to do so. The same holds true for other exercise-focused apps. Finding the right app means first deciding which problem the app should help solve.

A key finding of our systematic review is that the popularity of an app (with the stars awarded by users and the number of user ratings in the Google Play Store) is not a reliable indicator of its functional quality and/or potential for behavior change. Therefore, it is advisable to use app popularity as a starting point only when searching for an mHealth app. However, before making a choice, potential users should thoroughly evaluate the app’s ability to meet their specific needs.

### 4.3. Recommendations for Developers

There are some BCTs that are not often used in the development of mental health apps, that might further improve the effectiveness of the apps. *Assisting with or suggesting the restructuring of the physical environment* can have beneficial effects but is seldom used. The other two least-used techniques, *giving opportunity to plan for barriers* and *assisting with distraction or avoidance* may be harder to implement in the digital setting of an app, but are valuable additions to making sure users stick to the desired behavior.

Specific combinations of BCTs prove to be more effective than others (Dusseldorp et al., 2014). These synergies should be kept in mind when designing behavior change campaigns in apps, as they can severely enhance or hinder the effectiveness of the techniques used. While many apps already include the opportunity to *self-monitor behavior*, only a few include the possibility to *set goals*. This, paired with the possibility to *review, update and change goals*, as well as easily *grasp the differences in current actions and the goals set* can provide a strong motivator and should be included more often.

Finally, there seem to be other factors not included in the MARS and ABACUS that determine the popularity of an app. Further research should investigate this to find out exactly which factors contribute to the success of an app to give more detailed recommendations to developers.

## 5. Conclusions

The present study provides a systematic review of mental health mobile applications available on the Google Play Store, evaluating their quality and potential for behavior change. Using MARS and ABACUS, 77 apps were assessed for functional quality and their use of BCTs. Findings revealed that these apps generally displayed average quality, with engagement, functionality, esthetics, and information scores falling within a moderate range. Additionally, while most apps incorporated some BCTs, only a small number included a broad range of these techniques, limiting their potential for fostering substantial behavior change.

This study further explored whether MARS and ABACUS scores could predict app popularity, measured by stars awarded by users and number of user ratings. The results indicated that the scores had limited predictive power, suggesting that factors beyond app quality and BCTs influence user popularity. This emphasizes a need for additional research to explore alternative elements, such as marketing or user interface appeal, that are likely to impact app success.

This review highlights several recommendations for users and developers. For users, selecting an app that aligns closely with specific mental health goals is crucial, as app features vary widely. Developers are encouraged to integrate a wider array of BCTs and focus on holistic approaches that include physical activity, which was underrepresented in the reviewed apps.

Overall, this study underscores the importance of ongoing evaluation and rigorous testing of mental health apps to ensure they meet the needs of users and support effective behavior change. As mHealth continues to grow, further research will be essential to refine these tools and maximize their potential impact on mental health and well-being.

## Figures and Tables

**Table 1:** Descriptives—Mobile App Rating Scale (MARS).

	M	Md	SD	Min	Max
A—Engagement	3.67	3.60	0.55	2.40	4.60
B—Functionality	3.81	4.00	0.38	2.75	4.50
C—Esthetics	3.86	4.00	0.53	2.67	5.00
D—Information	3.42	3.40	0.52	1.50	4.33
E—Subjective Quality	2.99	3.00	0.85	1.25	4.75
Total	3.69	3.69	0.37	2.79	4.50

**Table 2:** The top 15 apps ranked according to their MARS score.

Name	Mean User Rating (Google Play Store)	MARS	ABACUS
MindDoc: Mental Health Support	4.30	4.50	12
Fabulous Daily Routine Planner	4.25	4.41	16
Amaha (Inner Hour): self-care	4.20	4.37	12
Lumiere: Ease Stress & Anxiety	4.60	4.33	11
How we Feel	4.60	4.25	12
Thinkable Mental Wellness	4.20	4.23	10
Daywell—Self Care Routine	4.30	4.16	12
Aware: Mindfulness & Wellbeing	4.25	4.11	12
Betwixt—The Mental Health Game	4.75	4.10	9
Smiling Mind: Meditation App	4.55	4.10	11
VOS Mental Health, AI therapy	4.45	4.10	16
COGITO (Neustart, MKT)	4.70	4.09	11
Panik Attack Help—Mind Ease	4.30	4.09	10
MyPossibleSelf: Mental Health	4.65	4.08	10
Emotions Diary and Mindfulness	4.60	4.05	11

**Table 3:** Descriptives—App Behavior Change Scale (ABACUS).

	M	Md	SD	Min	Max
1—Knowledge and Information	3.27	4.00	1.18	0	5
2—Goals and Planning	0.68	0.00	0.97	0	3
3—Feedback and Monitoring	3.13	3.00	1.31	0	7
4—Actions	2.23	2.00	0.83	0	6
Total	9.31	10.00	2.86	2	16

**Table 4:** The top 15 apps ranked according to their ABACUS score.

Name	Mean User Rating (Google Play Store)	MARS	ABACUS
Fabulous Daily Routine Planner	4.25	4.41	16
VOS Mental Health, AI therapy	4.45	4.10	16
Iona: Mental Health Support	4.65	4.02	15
Moodfit: Mental Health Fitness	4.25	3.86	14
Finch: Self Care Pet	4.80	3.83	14
Remente: Self Care, Wellbeing	4.15	3.80	14
CBT Companion: Therapy app	4.30	3.83	13
Stop Panic & Anxiety Self Help	4.35	3.55	13
MindDoc: Mental Health Support	4.30	4.50	12
Amaha (Inner Hour): self-care	4.20	4.37	12
How we Feel	4.60	4.25	12
Daywell—Self Care Routine	4.30	4.16	12
Aware: Mindfulness & Wellbeing	4.25	4.11	12
Intellect: Create A Better You	4.65	3.95	12
Rootd—Anxiety & Panic Relief	4.45	3.41	12

**Table 5:** Results of the regression model with MARS and ABACUS total scores predicting stars awarded by users in the Google Play Store.

Model Fit Measures							
				Overall Model Test			
Model	*R*	*R* ^2^	Adjusted *R*^2^	*F*	*df*1	*df*2	*p*
1	0.252	0.0635	0.0381	2.51	2	74	0.088

**Table 6:** Model Coefficients for regression model with stars awarded by users in the Google Play Store as dependent variable.

Model Coefficients—Mean User Ratings						
			95% Confidence Interval			
Predictor	Estimate	SE	Lower	Upper	*t*	*p*
Intercept	3.46210	0.4147	2.6358	4.2884	8.349	<0.001
MARS mean	0.25295	0.1404	−0.0268	0.5327	1.801	0.076
ABACUS mean	−0.00680	0.0183	−0.0432	0.0296	−0.372	0.711

**Table 7:** Model fit measures of the regression analysis with MARS and ABACUS as predictors for number of user ratings (Google Play Store).

Model Fit Measures							
				Overall Model Test			
Model	*R*	*R* ^2^	Adjusted *R*^2^	*F*	*df*1	*df*2	*p*
1	0.363	0.132	0.108	5.60	2	74	0.005

**Table 8:** Model coefficients of the regression with number of user ratings (Google Play Store) as dependent variable.

Model Coefficients—Number of User Ratings						
			95% Confidence Interval			
Predictor	Estimate	SE	Lower	Upper	*t*	*p*
Intercept	−85,368	91,488	−267,662	96,927	−0.933	0.354
MARS mean	7391	30,979	−54,336	69,118	0.239	0.812
ABACUS mean	8669	4036	628	16,710	2.148	0.035

## Data Availability

The raw data supporting the conclusions of this article will be made available by the authors on request.

## Supplementary Materials

The following supporting information can be downloaded at: https://www.mdpi.com/article/10.3390/ejihpe16010013/s1. References (Page et al., 2021a) are cited in the Supplementary Materials.
