# Supplementary material for: Supporting Mental Health with Apps: A Systematic Review of Potential and Quality of Implemented Behavior Change Techniques in Mobile Health Applications
Source: Eur J Investig Health Psychol Educ. 2026 Jan 14;16(1):13. doi: 10.3390/ejihpe16010013 (PMC12840162; doi:10.3390/ejihpe16010013)
Supplement: Supplementary file 1 [file ejihpe-16-00013-s001.zip › ejihpe-4011059 supplpmentary table .pdf]

Supplementary materials

I – List of keywords (English and German)

McKay et al. (2019b):

Social connection – Soziale Verbindung

Anxiety – Angst

Well-being - Wohlbefinden

Relaxation – Entspannung

Stress – Stress

Mood – Stimmung

Emotional intelligence – Emotionale Intelligenz

Empathy – Empathie

Loneliness – Einsamkeit

Resilience – Resilienz

Resilient – Resilient

Alsaity et al. (2022):

CBT – KVT

Cognitive Behavioral Therapy – Kognitive Verhaltenstherapie

Stoyanov et al. (2015):

Distress – Distress

New keywords:

Mental health – Mentale Gesundheit

Mental wellness – Mentale Wellness

Mental well-being – Mentales Wohlbefinden

II Results of the MARS subscales

Table S1

Descriptives for MARS subscale A – Engagement

	M	*SD*	*Min*	*Max*
A1 – Entertainment	3.42	0.75	2	5
A2 – Interest	3.71	0.73	3	5
A3 – Customization	3.36	0.90	1	5
A4 – Interactivity	3.75	0.86	1	5
A5 – Target group	4.10	0.42	3	5

Table S2

Descriptives for MARS subscale B – Functionality

	M	*SD*	*Min*	*Max*
B6 – Performance	3.94	0.57	2	5
B7 – Ease of use	3.78	0.60	2	5
B8 – Navigation	3.70	0.54	2	5
B9 – Gestural design	3.84	0.46	3	5

Table S3

Descriptives for MARS subscale C – Aesthetics

	M	*SD*	*Min*	*Max*
C10 – Layout	3.99	0.53	3	5
C11 – Graphics	3.92	0.70	3	5
C12 – Visual appeal	3.65	0.70	2	5

Table S4

Descriptives for MARS subscale D – Information

	N	*M*	*SD*	*Min*	*Max*
D13 – Accuracy of app description	77	4.04	0.55	2	5
D14 – Goals	50	2.86	0.57	2	4
D15 – Quality of information	66	3.70	0.58	3	5
D16 – Quantity of information	66	3.62	0.82	2	5
D17 – Visual information	15	4.00	0.38	3	5
D18 – Credibility	77	2.84	0.69	1	4
D19 – Evidence base	9	3.33	1.00	2	5

III Results of the ABACUS subscales
Table S5

Frequencies for ABACUS subscale 1 – Knowledge and Information

	Yes	No
1.1 Customizable	61	16
1.2 Created with expertise	58	19
1.3 Asks for baseline information	49	28
1.4 Instruction how to perform behavior	71	6
1.5 Provide information about (dis-)continuing behavior	13	64

Table S6

Frequencies for ABACUS subscale 2 – Goals and Planning

	Yes	No
2.1 Asks willingness for behavior change	8	69
2.2 Allows goal setting	24	53
2.3 Ability to review, update, and change goals	20	57

Table S7

Frequencies for ABACUS subscale 3 – Feedback and Monitoring

	Yes	No
3.1 Gives ability to understand difference between current action and goals	25	52
3.2 Allows self-monitoring behavior	74	3
3.3 Ability to share behavior with others	18	59
3.4 Gives feedback (personal or automatic)	35	42
3.5 Ability to export data	17	60
3.6 Offers material or social reward/incentive	5	72

3.7 Provides general encouragement	67	10

Table S8

Frequencies for ABACUS subscale 4 – Actions

	Yes	No
4.1 Offers reminders, prompts or cues for activity	70	7
4.2 Encourages positive habit formation	73	4
4.3 Allows or encourage activity beyond daily activities	21	56
4.4 Provides opportunity to plan for barriers	2	75
4.5 Assists with or suggests restructuring of physical or social environment	4	73
4.6 Assists with distraction or avoidance	2	75

IV – List of all apps with MARS and ABACUS scores (sorted alphabetically)

Name	Mean rating (Google Play)	MARS	ABACUS
5 Minute Journal: Self-Care	4.55	3.76	8
6000 thoughts | AI Life Coach	4.00	3.53	8
Action for Happiness: Get Tips	3.90	3.05	4
Amaha (Inner Hour): self-care	4.20	4.37	12
Amaru: The Self-Care Pet	4.70	3.38	9
Amobear: Mood Tracker	4.60	2.79	4
Anxiety & Stress Log, Analysis	4.10	3.69	5
Anxiety Relief Apps & Hypnosis	4.40	3.02	2
Anxiety Tracker & Self Care	4.30	3.05	6
Aura: Meditation & Sleep, CBT	4.45	3.88	8
Aware: Mindfulness & Wellbeing	4.25	4.11	12
Awarefy - CBT & AI Therapy	3.30	3.83	11
being: self therapy & CBT ai	3.60	3.35	8
BetterMe: Mental Health	3.85	3.58	8
Betwixt - The Mental Health Game	4.75	4.10	9
Bipolar UK Mood Tracker	4.50	3.72	7
Blissful Journal, Mood Tracker	4.55	3.37	6
CBT Companion: Therapy app	4.30	3.83	13
CBT Thought Diary: Depression	4.20	3.20	5
CBT Tools for Healthy Living	4.50	3.51	10
Chiku - Journal & Mood Tracker	4.35	3.85	10
Cingulo - Mental Wellness	4.85	3.95	10

Clarity - CBT Thought Diary	4.10	3.60	7
Clear Minds: Meditation, Relax	3.75	3.10	7
COGITO (Neustart, MKT)	4.70	4.09	11
Dare: Anxiety & Panic Attacks	4.70	3.62	8
Daywell - Self Care Routine	4.30	4.16	12
DBT Coach : Guided Therapy	3.95	3.57	10
Don't panic	4.45	3.22	8
Emotions Diary and Mindfulness	4.60	4.05	11
Evolve: Self-Care & Meditation	4.55	4.00	11
Fabulous Daily Routine Planner	4.25	4.41	16
FearTools - Anxiety Aid	4.40	3.51	7
Finch: Self Care Pet	4.80	3.83	14
Gladdy: Diary, Mental Practice	4.10	3.06	5
HappierMe: Master your Mind	4.30	3.64	9
How we Feel	4.60	4.25	12
I'm Fine: Mental Health Guide	3.30	3.46	9
Innergy: Meditation & Wellbeing	4.60	3.45	9
Intellect: Create A Better You	4.65	3.95	12
Iona: Mental Health Support	4.65	4.02	15
Lumiere: Ease Stress & Anxiety	4.60	4.33	11
Me: Reflect for Self Awareness	4.60	3.65	10
Mind Reset - Just 2 min a day!6.4.8	4.20	3.29	5
Mind Tracker	4.45	3.29	6
Mindberg: Jungian Psychology	4.50	3.62	9
MindDiary: Mood Tracker	4.40	3.48	6

MindDoc: Mental Health Support	4.30	4.50	12
MindFi: Mind Fitness for All	4.00	3.58	10
MINDSET by Dive Studios	4.80	3.88	10
Mintalitea - Mental Health CBT	4.20	3.18	5
Moodfit: Mental Health Fitness	4.25	3.86	14
Mooditude: Mental Health App	4.30	3.68	11
MoodTools - Depression Aid	4.25	3.89	6
My Stress Diary - Mood Antist	3.90	3.85	10
MyPossibleSelf: Mental Health	4.65	4.08	10
Panik Attack Help - Mind Ease	4.30	4.09	10
Parazute: Mental journal	4.40	4.00	10
Psychology & Wellbeing: Relate	4.70	3.31	9
Remente: Self Care, Wellbeing	4.15	3.80	14
Rootd - Anxiety & Panic Relief	4.45	3.41	12
SELF: Self Care & Self Love	4.70	3.90	11
Self-Esteem and Confidence	4.45	3.30	6
Sintelly	4.35	3.92	11
Smiling Mind: Meditation App	4.55	4.10	11
Stop Panic & Anxiety Self Help	4.35	3.55	13
Stress & Anxiety: Head On	3.70	3.53	10
The Self Compassion App	4.40	3.85	10
Thinkable Mental Wellness	4.20	4.23	10
Tochi - Mood Tracker, Journal	4.45	3.26	6
UpLife: Mental Health Therapy	4.40	3.65	8
Voidpet Garden: Mental Health	4.70	3.90	11

VOS Mental Health, AI therapy	4.45	4.10	16
What's Up? Mental Health App	4.15	3.43	7
WorryTree: Anxiety Relief CBT	4.00	3.81	9
Wysa: Anxietyy, therapy chatbot	4.55	3.93	10
Youper - CBT Therapy Chatbot	3.85	3.15	10
